# Distinguishing core from penumbra by lipid profiles using Mass Spectrometry Imaging in a transgenic mouse model of ischemic stroke

**DOI:** 10.1038/s41598-018-37612-5

**Published:** 2019-01-31

**Authors:** I. A. Mulder, N. Ogrinc Potočnik, L. A. M. Broos, A. Prop, M. J. H. Wermer, R. M. A. Heeren, A. M. J. M. van den Maagdenberg

**Affiliations:** 1 Department of Neurology, Leiden University Medical Center, 2300 RC Leiden, The Netherlands; 2 Maastricht MultiModal Molecular Imaging (M4I) Institute, Division of Imaging Mass Spectrometry, Maastricht University, 6229 ER Maastricht, The Netherlands; 3 Department of Human Genetics, Leiden University Medical Center, 2300 RC Leiden, The Netherlands

## Abstract

Detecting different lipid profiles in early infarct development may give an insight on the fate of compromised tissue. Here we used Mass Spectrometry Imaging to identify lipids at 4, 8 and 24 hours after ischemic stroke in mice, induced by transient middle cerebral artery occlusion (tMCAO). Combining linear transparency overlay, a clustering pipeline and spatial segmentation, we identified three regions: infarct core, penumbra (i.e. comprised tissue that is not yet converted to core), and surrounding healthy tissue. Phosphatidylinositol 4-phosphate (*m/z* = 965.5) became visible in the penumbra 24 hours after tMCAO. Infarct evolution was shown by 2D-renderings of multiple phosphatidylcholine (PC) and Lyso-PC isoforms. High-resolution Secondary Ion Mass Spectrometry, to evaluate sodium/potassium ratios, revealed a significant increase in sodium and a decrease in potassium species in the ischemic area (core and penumbra) compared to healthy tissue at 24 hours after tMCAO. In a transgenic mouse model with an enhanced susceptibility to ischemic stroke, we found a more pronounced discrimination in sodium/potassium ratios between penumbra and healthy regions. Insight in changes in lipid profiles in the first hours of stroke may guide the development of new prognostic biomarkers and novel therapeutic targets to minimize infarct progression.

## Introduction

Ischemic stroke is a severe neurological event and its incidence in Western society is increasing^1^. Ischemic stroke generally occurs due to an interruption of blood supply, drastically decreasing glucose and oxygen concentrations within seconds. ATP levels drop rapidly, resulting in random ion leakage across the neuronal cell membrane. As a consequence, the ion balance in the tissue is severely disrupted, ultimately leading to cerebral ischemia. The ischemic territory consists of a core (necrotic tissue) that is surrounded by a border zone, the “penumbra”. Penumbra tissue has only partially reduced blood flow, just above the ischemic threshold level^2^. When the ischemia is not resolved in time, the penumbra will be converted to core tissue. The mechanisms involved in this transition are only partly known^3–7^.

During the ischemic event, different lipid pathways are activated by multiple lipids (for example phosphatidylcholine (PC) and Lysophosphatidylcholine (LPC)) and second messenger molecules that can act in a neuroprotective or neurodegenerative way^6^. Identification of the molecular species in the different regions of the ischemic area, especially in the early stages of infarct development, is crucial to identify the molecular mechanisms that may determine the fate of the penumbra.

Mass Spectrometry Imaging (MSI) is a powerful technique for identifying hundreds of molecules simultaneously within a single tissue section in a histological context^8,9^. This technique is particularly useful to evaluate lipid distributions on tissue sections for which there are generally no antibodies available. MSI, specifically Matrix-Assisted Laser Desorption Ionization (MALDI), has been applied in neurodegenerative diseases, including research on the distribution of Aβ peptides co-localizing with different elements^10,11^, neuropeptides in Parkinson’s disease^12^ and global changes in phospholipids in a transgenic mouse model for Alzheimer’s disease^13^. MSI offers the unique capability to simultaneously detect pathological hallmarks and related biomolecular patterns of neurodegeneration, making it a powerful monitoring tool for disease pathogenesis and prognosis.

Here, we used MSI to assess changes in lipids between the penumbra and infarct core during the early stroke evolution in mice. Until now, studies have focussed on the distribution of phospholipids in the ischemic region compared to the healthy surrounding area of the brain and/or after 24 hours of ischemic stroke induction^3–7,14^. It was demonstrated that in the ischemic region Na^+^ is elevated with a concomitant reduction of K^+^ ^3,5,6^. As large changes in alkali cation concentrations influence the ionization efficiency of phospholipids, matrix effects may have confounded those results. Later studies reduced the matrix effect, by either desalting the tissue sections prior to MALDI or using an internal standard as a normalization factor, and revealed different distributions of phospholipids^14,15^. An increase of LPC (16:0) was observed in the ischemic region with concomitant membrane degradation of free fatty acids and the degradation of PCs into LPCs^4,6^.

We assessed MALDI-MSI derived lipid profiles in three regions of interest (core, penumbra and healthy surrounding tissue) at three time points (4, 8 and 24 hours) after transient middle cerebral artery occlusion (tMCAO) in mice. Additionally, we included a transgenic mouse model that expresses mutant hyperactive voltage-gated Ca_V_2.1 Ca^2+^ channels that cause Familial Hemiplegic Migraine type 1 (FHM1)^16^. These mutants are particularly interesting for this study because a higher cerebral blood flow is required for tissue survival after tMCAO in these mice. Therefore, infarction already occurs in these mutants with milder ischemia compared to wild-type mice, which may also occur in patients and thereby increasing the translational value of our study. With this approach, we aimed to study in detail the regional lipid distribution in early ischemic stroked development.

## Results

### Linear transparency overlay, clustering pipeline and spatial segmentation reveal the penumbra and core in ischemic area

**Figure 1:**
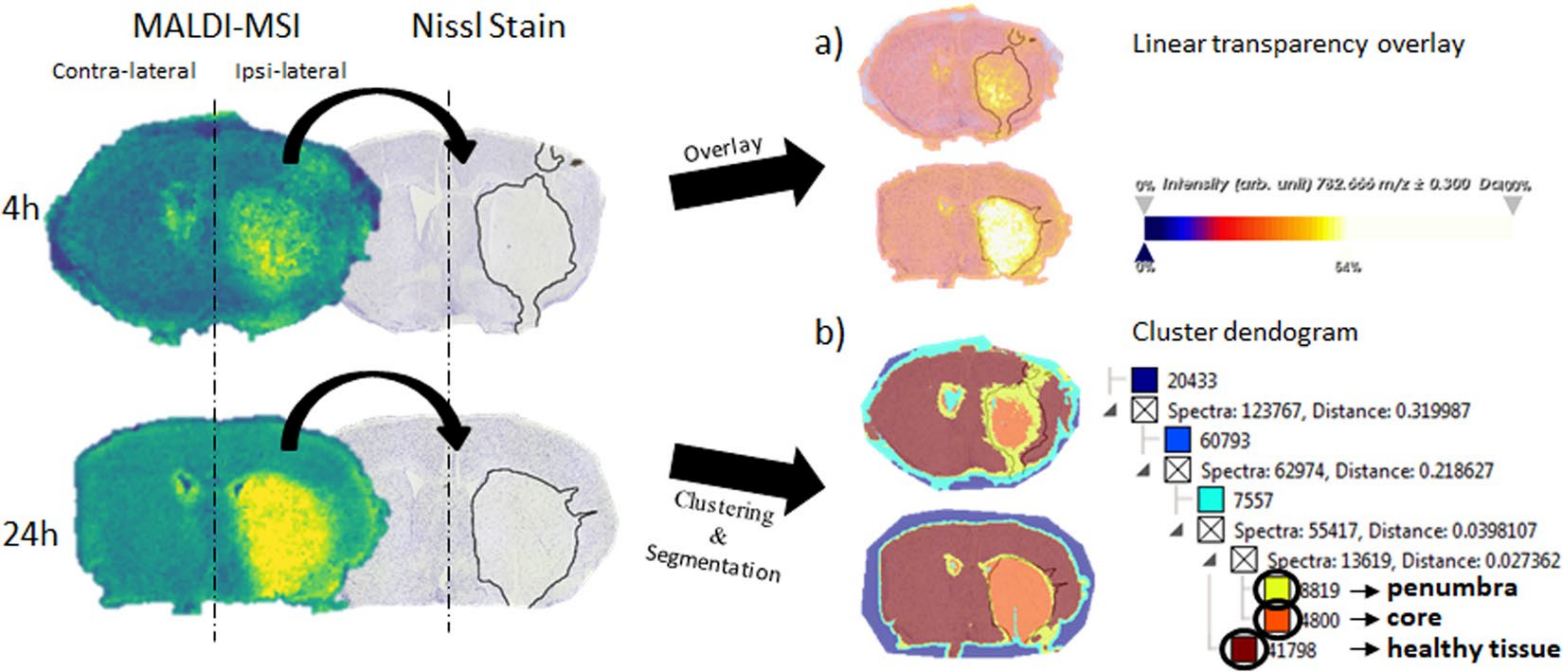
A schematic representation of the manual overlays of MALDI-MSI and Nissl stain with marked necrotic border in SCiLS Lab 2016b. The manual overlay was followed by a linear transparency overlay (**a**) of a selected *m/z* value (in the example *m/z* = 782.6 ± 0.3). The high intensity of the [PC(34:1) + Na]^+^ perfectly aligns within the necrotic border of the Nissl stain. (**b**) In the next step, the manual overlay was followed by a spatial segmentation pipeline built in the software. Once the segmentation map was created it detected prominent spatial regions ipsilateral, representing core, penumbra and healthy tissue, here at 4 hours after transient middle cerebral artery occlusion.

We used a histology-directed spatial segmentation approach (Fig. 1), employed to segment sections into ipsi- (ischemic) and contra-lateral (healthy) sides. This technique was combined with manual linear transparency overlay. This approach allowed us to successfully co-register the selected *m/z* values in positive ion mode with the Nissl-stained sections, annotated with the necrotic border. All of the selected ions perfectly align within the necrotic border, separating the ischemic region from the rest of the tissue (Fig. 1a). The software-derived spatial segmentation pipeline revealed a further segmentation of the positive ion mode data into ipsi-lateral sub-regions, i.e. core, penumbra and surrounding healthy tissue, which can’t be solely observed by histological staining. Prominent spatial regions are visualized already at 4 hours after tMCAO, as can be observed in the example of Fig. 1b.

### Lipid profiles in core and penumbra in the acute phase of ischemic stroke

**Figure 2:**
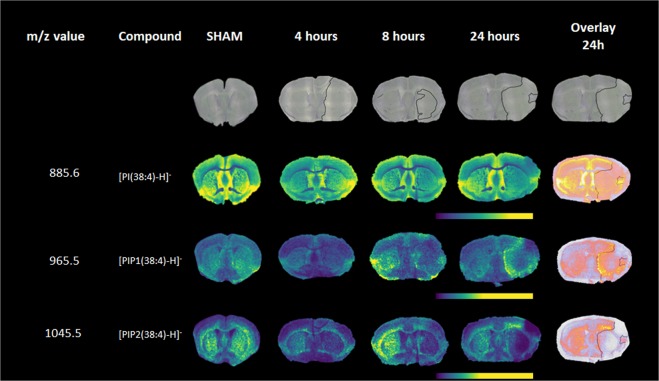
MALDI images showing the distribution of the PIP molecular family and accompanying Nissl stainings. Selected MALDI *m/z* images acquired at 50-µm spatial resolution showing the distribution of specific lipids *m/z* = 885.6 [PI(38:4) − H]^−^, *m/z* = 965.5 [PIP(38:4) − H]^−^, and *m/z* = 1045.5 [PIP_2_(38:4) − H]^−^ in a SHAM WT mouse and at 4, 8 and 24 hours after transient middle cerebral artery occlusion measured in negative ion mode. The last column represents the linear transparency overlays of *m/z* = 885.6 [PI(38:4) − H]^−^, *m/z* = 965.5 [PIP(38:4) − H]^−^, and *m/z* = 1045.5 [PIP_2_(38:4) − H]^−^ with Nissl staining at 24 hours after tMCAO.

The segmented regions of the infarct were investigated by first manually drawing ROIs with the same number of pixels (Supplementary Fig. S1a). In the positive ion mode mean spectra we observed an increase in relative intensities of *m/z* = 756.6 [PC(32:0) + Na]^+^ and 810.6 [PC(36:1) + Na]^+^, while *m/z* = 772.6 [PC(32:0) + K]^+^ shows a decrease in the core region compared to surrounding penumbra and healthy tissue at 4 hours post tMCAO. There are no relative intensity changes observed at *m/z* = 760.6 [PC(32:0) + H]^+^ (Supplementary Fig. S1b). The same pattern is observed when box plotting intensity variations of these ions (n = 3). In Supplementary Fig. S1c we show a similar variation in the healthy (ipsi and contra) and penumbra. However, a slight increase of variation in the core region for *m/z* = 756.6 is also shown, while *m/z* = 810.6 (Supplementary Fig. S1d) shows a gradual increase from healthy to the core. In contrast, *m/z* = 772.6 shows a larger decrease in the penumbra and core. The variation of signal intensity at *m/z* = 760.6 is constant throughout the ROIs. While investigating the linear transparency overlays with selected lipid signals in negative ion mode, a clear difference was observed between the core and penumbra for *m/z* = 965.5 and *m/z* = 1045.5. The selected molecular species, specifically *m/z* = 965.5, showed an increase in signal intensity (increasing over time), but notably only in the border zone surrounding the core (Fig. 2). A decrease in signal intensity was observed in the infarct core at 4 hours that was more pronounced at 8 hours after the stroke induction. MS/MS analysis in negative ion mode identified *m/z* = 965.5 ion as [PIP(38:4) − H]^−^ (phosphatidylinositol 4-phosphate) (Supplementary Fig. S3) and [PIP_2_(38:4) − H]^−^phosphatidylinositol 4,5-bisphosphate, (*m/z* = 1045.5) (Supplementary Fig. S4). Additional MS/MS of [PI(38:4) − H]^−^ (phosphatidylinositol) was taken for comparison (Supplementary Fig. S2). The fragmentation spectra of all three PI and PIP species have been consistent with the previously observed data^17,18^.

### Lipid profiles in ischemic stroke evolution in core and penumbra

**Figure 3:**
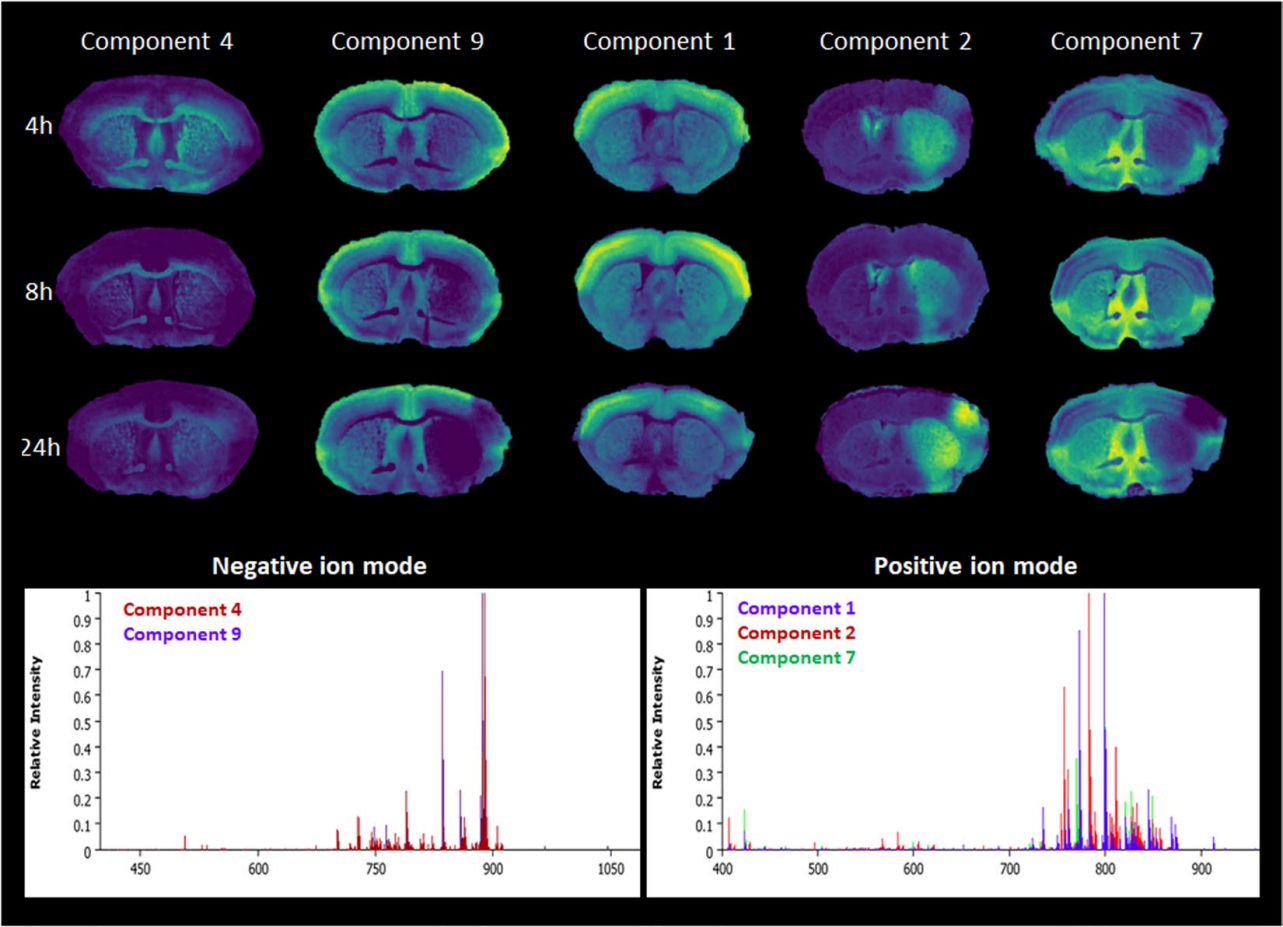
Probabilistic latent semantic analysis (pLSA) loading plots and spectra of resulting components of the WT mice in negative (components 4 and 9) and positive (components 1, 2 and 7) ion mode 24 hours after transient middle cerebral artery occlusion.

The same segmentation pipeline was used to investigate lipid profiles in the spatial regions across the three time points of stroke evolution. Probabilistic latent semantic analysis (pLSA) was used for the statistical analysis of data of the spatial regions (using the described spatial segmentation) (Fig. 3). The data obtained for negative and positive ion modes was best described with 7 and 10 components. The components with the clearest difference between the ipsi- and contra-lateral side are shown in Fig. 3. Components 4 and 9 exhibited the most marked difference in negative ion mode; components 1, 2 and 7 were most distinctive in positive ion mode. In negative ion mode the largest contributions to component 9 came from the *m/z* = 885.6 [PI (38:4) − H]^−^, which is known to differentiate grey from white matter. White matter was predominantly characterized by *m/z* = 834.6 PS(40:6) at (component 4). The negative mode ions that attributed most to the border zone of the ischemia are *m/z* = 909.6, 833.6 for component 4 and *m/z* = 965.6 [PIP(38:4) − H]^−^,1045.5 [PIP_2_(38:4) − H]^−^ for component 9. Changes in these compounds were visible already at 4 hours after tMCAO and increased up to 24 hours. The box intensity plots (Supplementary Fig. S5) for [PIP(38:4) − H]^−^ show a larger variation of intensity at 4 hours and 8 hours in healthy ipsi- and contralateral tissue and a decrease in the penumbra and core. The biggest variation is observed at 24 hours in the penumbra. For [PIP_2_(38:4) − H]^−^ there is a bigger difference in relative intensities between the healthy tissue and between penumbra and core. At 24 hours the intensity variations in the penumbra become similar to the healthy tissue opposed to the core.

We also revealed the presence of cardiolipin (CL) lipids (found in the inner mitochondrial membrane and play a coordinating role in apoptotic cell death^19^) and ganglioside (GM) lipids (found in the outer layer of the plasma membrane and important in neuronal function and cell death^7,20^), although no changes with respect to spatial or temporal distribution relevant to ischemic infarct evolution were observed (Supplementary Table S2).

**Figure 4:**
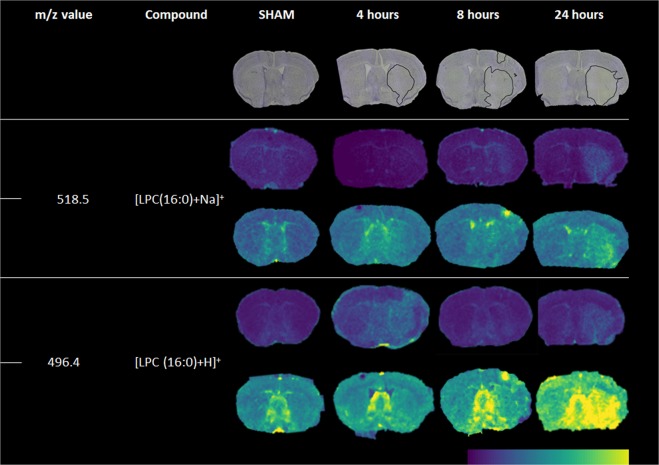
MALDI images showing the distribution of lyso-lipids and accompanying Nissl stainings. Selected MALDI *m/z* images acquired at 50-µm spatial resolution showing the distribution of lyso-lipids in a SHAM WT mouse and at 4, 8 and 24 hours after transient middle cerebral artery occlusion measured in positive ion mode. Each row of the selected species represents un-washed (top) and washed (bottom) tissue section.

In positive ion mode, components revealed a clearer differentiation between the ischemic and non-ischemic hemispheres. Specifically, within the ipsi-lateral hemisphere, components 2 and 7 showed molecular profiles that correlated with the penumbra and core of the ischemia. Lipid species, *m/z* = 756.6 [PC (32:0) + Na]^+^ and *m/z* = 782.6 [PC (34:1) + Na]^+^ show high abundancy in the ischemic area compared to the lipid profile in the non-ischemic hemisphere. An increase of lyso-lipid intensities in the ischemic area, mainly [LPC (16:0) + H]^+^ (*m/z* = 496.4) and [LPC (16:0) + Na]^+^ (*m/z* = 518.5) (Fig. 4), can be induced by washing the tissue sections prior to MALDI analysis, which is in line with previous data^4^. The intensities of the LPC species seem to increase in the course of 24 hours (Fig. 4). In respect to the images, similar tradelines are observed for box intensity plots (n = 3) (Supplementary Fig. S5). The variation of intensities increase over the course of 24 hours for [LPC (16:0) + H]^+^ and [LPC (16:0) + Na]^+^ in the core. The same pattern is observed for the rest of the selected [PC+ Na]^+^ species in contrast to [PC (32:0) + K]^+^. MALDI images of additional lipid species in positive ion mode (washed and unwashed) at 4, 8 and 24 hours after tMCAO in WT mice can be found in Supplementary Table S1.

To quantify the relative changes of prominent lipid species, we calculated fold changes as the mean ratio of signal intensity between ischemic (core, penumbra) and healthy ROIs (n = 3) at 4, 8 and 24 hours after tMCAO (Supplementary Fig. S7) with 95% confidence interval. Fold changes were expressed for S7 a) [LPC (16:0) + H]^+^ *m/z* = 496.4, S7b) [LPC (16:0) + Na]^+^ *m/z* = 518.5, S7c) [PC(32:0) + Na]^+^ *m/z* = 756.6, S7d) [PC(32:0) + K]^+^ *m/z* = 772.6 in positive ion mode and S7e) [PIP (38:4) − H]^−^ and S7f) [PIP_2_ (38:4) − H]^−^ in negative ion mode. Complementing previously observed data the largest increase of [LPC (16:0) + H]^+^ and [LPC (16:0) + Na]^+^ was observed in the core at 24 hours post tMCAO. Interestingly, the washed tissue didn’t show a significant increase at 24 hours post tMCAO for both selected species, though more pronounced in the MALDI ion images. [PC(32:0) + Na]^+^ and [PC(32:0) + K]^+^ show their respective fold increase/decrease in the core. The biggest difference is observed in the [PIP (38:4) − H]^−^ ion. At 4 hours and 8 hours there is a fold decrease in the penumbra, while at 24 hours there is a clear increase in the penumbra. Similarly, to the MALDI images there is a significant decrease of the [PIP_2_ (38:4) − H]^−^ in the core and penumbra starting at 8 hours post tMCAO.

### Sodium/potassium ratio’s in the early phase of stroke evolution

**Figure 5:**
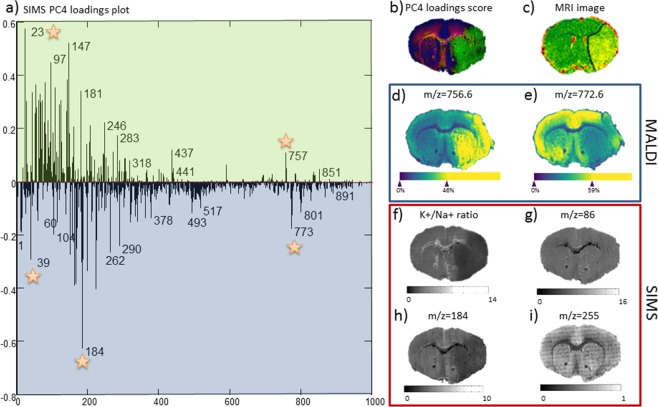
MRI, SIMS and MALDI images revealing the distribution of the infarct core in wild-type mice after transient middle cerebral artery occlusion. (**a**) PC4 loadings plot revealing the distribution of co-related species with Na^+^ (positive loadings plot-green) and K^+^ (negative loadings-blue), (**b**) RGB overlay of three PCs (red; PC-3, green; PC4, and blue; PC-4); (**c**) T2-weighted MRI image with delineated infarct border; MALDI image of (**d**) *m/z* = 756.6 [PC(32:0) + Na]^+^ and (**e**) *m/z* = 772.6 [PC(32:0) + K]^+^; (**f**) K^+^/Na^+^ ratio, (**g**) choline fragment, (**h**) phosphatidylcholine fragment in positive ion mode and (**i**) palmitic acid (16:0) *m/z* = 255, in ischemia 24 hours after transient middle middle cerebral artery occlusion.

As the sodiated/potassiated species may change over the course of 24 hours after tMCAO, we performed high-resolution Secondary Ion Mass Spectrometry (SIMS) analysis to evaluate the Na^+^/K^+^ ratio. Principal component analysis was performed to determine the distribution of Na^+^ (*m/z* = 23) and K^+^ (*m/z* = 39) species and related molecular species. PC4 loadings plot (Fig. 5a) described with 0.293% variance reveals the distribution of species related with Na^+^ (positive loadings plot; green) and K^+^ (negative loadings; blue). The distribution of Na^+^ and K^+^ respective related species is shown in the RGB image score overlay of PC + 4 (green), PC − 4 (blue) and PC3 (red) (Fig. 5c). SIMS images of specific lipids, such as *m/z* = 756.6 [PC(32:0) + Na]^+^ and *m/z* = 772.6 [PC(32:0) + K]^+^, show a distribution similar to the acquired MALDI images (Fig. 5d and e) of consecutive sections. PC + 4 reveals a significant increase in Na^+^ and related species in the ischemic area opposed to the surrounding healthy tissue (Fig. 5c). The distribution of K^+^ and related species shows a slight decrease in the ischemic region (Fig. 5b), which is also observed when plotting the ratio between Na^+^ and K^+^ (Fig. 5f). The distribution of choline (*m/z* = 86) and phosphatidylcholine fragments (*m/z* = 184) is shown in Fig. 5g/h. Even though choline has a very homogeneous distribution across regions, the phosphatidylcholine fragments show a slight depletion in the ischemic core. In negative ion mode, fatty acids palmitic acid (16:0) *m/z* = 255, exhibit a homogeneous distribution across regions (Fig. 5i), very similar to the choline fragment.

### Lipid composition in a transgenic mouse model with increased stroke vulnerability

**Figure 6:**
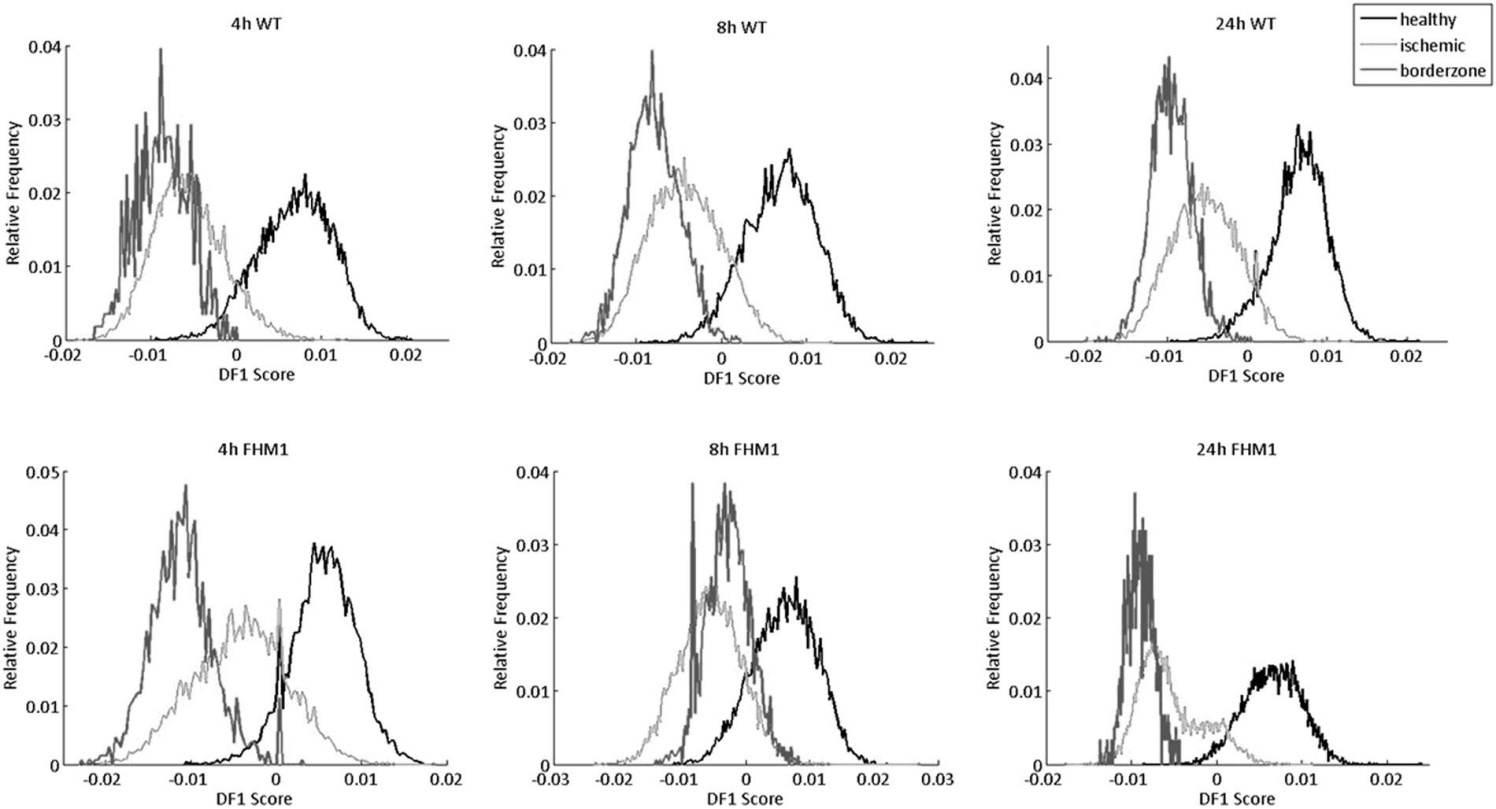
Linear discriminant analysis histograms of wild-type (top row) and transgenic FHM1 mice (bottom row) of segmented ROI areas (healthy (black), ischemic (light grey) and borderzone (dark grey)) at 4, 8 and 24 hours after transient middle cerebral artery occlusion.

Next, we assessed lipid profiles in a transgenic mouse model in which a higher cerebral blood flow is required for tissue survival after tMCAO, so the penumbra in these mutant mice is more easily converted to core tissue^21^. In Fig. 6 the histograms of measurements in positive ion mode are shown for the different brain regions of wild-type (WT) and FHM1 mutant mice at 4, 8 and 24 hours after tMCAO. In FHM1 mutant mice a more pronounced discrimination between the penumbra and healthy tissue was observed compared to WT mice. Moreover, the largest change in distribution patterns is seen in the penumbra in mutant mice at all time points as well as compared to the WT mice (Fig. 6). The scaled loadings (Supplementary Fig. S4) show the contribution of each individual molecular mass to the differentiation observed in the histograms between the healthy, penumbra and ischemic regions. The loadings plots indicate that the major difference corresponds mainly to the [M + Na]^+^ and [M + K]^+^ ions of the same lipid species.

## Discussion

High-resolution SIMS imaging combined with rapid lipid detection with MALDI-MSI is able to identify molecular profiles that can distinguish the ischemic and non-ischemic (healthy) regions after experimentally induced stroke. Data processing with linear transparency overlays and spatial segmentation made it possible not only to differentiate between ischemic and healthy mouse tissue already 4 hours after stroke induction, but also between infarct core and penumbra, which are not distinguishable at that time point with Nissl-stained images.

The most prominent difference between the ischemic and non-ischemic areas is governed by the Na^+^/K^+^ availability for the ionization of the molecular species. Lipid profiles revealed an extensive increase in Na^+^ species, both from the penumbra to the core and during stroke development. The distribution of Na^+^ and K^+^ species shows a specific pattern: where Na^+^ species are high, species of K^+^ are low, and *vice versa*. This suggests that the intensity of sodiated “pseudo-molecular” ions, for example [M + Na]^+^, is dependent on the level of sodium ions present in the tissue, (*idem* for potassium or other ions). A plausible explanation is the massive failure of sodium/potassium pumps within the ischemic area. This failure results in relative increase in sodium and decrease in potassium concentrations inside the cell due to passive diffusion influx of Na^+^ and efflux of K^+^ due to intracellular versus extracellular concentration and osmolarity differences^4,22^. The relative high intracellular levels of Na^+^ ions lead to the production of [M + Na]^+^ pseudo-molecular ions during the ionization process. Combined with competition between K^+^ and Na^+^ ions for the same molecular species during the ionization, this will result in an *apparent* increase in detected [M + Na]^+^ signal and an *apparent* reduction in the detected signals of [M + K]^+^. This would imply that lipid species that are related to the Na^+^/K^+^ ratio should *not* be considered as reliable biomarkers when studying ischemia.

Overall, [PC + Na]^+^ showed an increase in the ischemic core whereas [PC + K]^+^ showed an overall decrease, a finding which is in line with previous data in rats^4^. No clear changes in the ischemic or healthy tissue were found with respect to the PC [M + H]^+^ concentration. The same was true for [LPC (16:0) + Na]^+^ (*m/z* = 518.5), which was increased in the ischemic area, starting a few hours after tMCAO, and visible as a difference between the various ischemic regions at 8 hours after tMCAO (Figs 4, S5 and S7b). These results confirm findings in a study in rat pups where sodium adduct of LPC (16:0) was present in the ischemic area but not in the healthy tissue at 24 and 48 hours after induction of the infarct^4^. LPCs have a relatively short half-life and are formed, together with fatty acids, as metabolic intermediates, during the breakdown of other lipids. LPCs increase as part of a neuroinflammatory response; an increased activation of PLA_2_ eventually results in increased neuronal damage^6,22^. This could explain the observed decreased intensity of *m/z* = 184 fragment ion in the infarct core.

During neuronal cell death, cell membranes become more permeable, extracellular Na^+^ and Ca^+^ enter the cell and glutamate is released, causing activation of NMDA receptors. This triggers multiple cascades such as peri-infarct depolarizations spreading in the penumbra and activation of multiple phospholipases like PLA_2_ resulting in increased amounts of phosphatidylinositol (PI) in the borderzone. Additionally, second messenger lipids are released (1,2-diacylglycerol, (Lyso-)PA, (Lyso-)PC+ Na^+^). The latter, on its turn, activates PLA_2_ and the destructive circle continues, turning the penumbra further into necrotic core^6,22^.

A difference between the penumbra and the core region in the negative ion mode MALDI images was observed. Specifically, the distribution of *m/z* = 965.5 ion, identified as [PIP (38:4) − H]^−^ showed an increased signal intensity in the penumbra surrounding the core area 24 hours after tMCAO. The more abundant decline of PIP_2_ compared to PIP in the infarct core is in line with data of several animal models for global and focal ischemia^23–28^. The signal transduction pathway involving hydrolysis of polyphosphoinositides (poly-PI) in the central nervous system is largely responsible for the control of the intracellular calcium homeostasis, as well as receptor activation via neurotransmitters, such as glutamate, acetylcholine, dopamine and serotonin^28^. Sustaining receptor-mediated Poly-IP signalling activity is highly energy-consuming, requiring several moles of ATP via PI and PIP kinases^28^. With the depletion of ATP during ischemia, PIP_2_ high-energy dependent turnover from PIP might be abolished, which could be the reason for the increased PIP concentration compared to PIP_2_ in the penumbra at 24 hours. Also, stimulation of the poly-PI pathway in the border zone might be part of an early event resulting in ischemia, which is also suggested by the rapid and transient increase of Ins(1,4,5)P after global cerebral ischemia^28^. These results suggest that the change in poly-PI levels is important for the fate of penumbra tissue. PIP and PIP_2_ levels, therefore, may serve as a signature biomolecular pattern to identify whether penumbra tissue is about to become necrotic.

The molecular distribution of cardiolipin and ganglioside were also investigated, since they both play an important role in the apoptotic signalling pathway^7,19,20^. CL has been shown to be affected by post-traumatic injury^7,29–31^. Catabolism of cardiolipin occurs by the catalysis of phospholipase A2 (PLA_2_). However, CL distribution patterns do not seem to be changed in the ischemic core in the early time course after tMCAO. One explanation could be that early after induction of ischemia (minutes-hours), necrosis has the upper hand, whereas apoptosis comes into play at a later stage of infarct development (hours-days)^32^. This time-dependant activation of different pathways leading to cell death has previous been shown in large detail^33^. GM, as part of the plasma membrane^34^, has been shown to ameliorate deficit after stroke^35,36^. Here we did not find a clear difference in GM-accumulation or degradation in the ischemic core or penumbra compared to the healthy tissue. We speculate that this process is activated at a later stage of infarct evolution.

In ischemic tissue of the transgenic mutant, at 24 hours after tMCAO, Na^+^ and K^+^ species seem to contribute more to the difference compared to WT mice. We hypothesize that this difference is due to a higher abundancy of sodium or potassium across the ischemic region in the mutant mice. For all three time points, especially at 24 hours after tMCAO, the levels of sodiated species in the border zone are higher in mutant compared to WT mice.

In conclusion, the infarct evolution was clearly shown by 2D-renderings of multiple PC and LPC isoforms. The penumbra became clearly distinguishable from the core at the 8-hour time point after tMCAO, as evidenced by differences in the levels of PIP and PIPI_2_. These lipids, therefore, could serve as early ischemic markers. Further research is needed to investigate whether or not increased levels of PIP and PIP_2_ in combination with the ratio between both could indicate if the compromised penumbra will convert into core tissue or not.

## Materials and Methods

### Animal protocol

All animal experiments were approved by the Animal Experiment Ethics Committee of Leiden University Medical Center and all experiments were performed in accordance with relevant guidelines and regulations. Male FHM1 mutant mice and their WT littermates, age 3.5 months ± 2 weeks, were used.

Experimental stroke was induced using the widely used tMCAO model^37^. Isoflurane (3% induction, 1.5% maintenance) in 70% pressurized air and 30% O_2_ was used as anaesthetic. Additional, Carprofen 5 mg/kg, s.c. (AST Farma B.V.) was given before surgery. During surgery the mouse body temperature was maintained at ~37 °C using a feedback system. The MCA was occluded using an intraluminal silicone-coated nylon monofilament (Doccol cooperation), for 30 minutes. Thereafter the filament was removed to allow for reperfusion. After surgery, mice were allowed to wake up in a temperature-controlled incubator (Peco Services Ltd) kept at ~33 °C for 2 hours with easy access to food and water. On a subset of animals, SHAM surgery was performed using the same protocol, only without insertion of the filament.

### Magnetic resonance imaging

A subset of animals were scanned 24 hours after tMCAO surgery, to evaluate the infarct region *in vivo*, using a small-animal 7 T MRI system (Bruker BioSpin) under isoflurane anesthesia. A Multi Slice Multi Echo (MSME) sequence protocol was run with TR/TE of 4.000 ms/9 ms, 20 echoes, 2 averages, matrix 128 × 128 mm, FOV of 2.50 cm, bandwidth 59523.8 Hz, slice thickness of 0.5 mm and 16 slices (no gap). Quantitative T2 maps were calculated using Paravision 5.1 software (Bruker BioSpin).

### Sacrifice and histology

Per time point, 4 mice were sacrificed at 4, 8 and 24 hours after tMCAO and at 24 hours after SHAM surgery using the *in situ* funnel-freezing technique^38^ because this technique limits molecular post-mortem degradation as much as possible compared to other existing sacrificing techniques. In brief, under deep anaesthesia (3% Isoflurane in 70% pressurized air and 30% O_2_), the brains were frozen *in vivo* using liquid nitrogen. Using a funnel placed onto the skull, with the posterior rim at the lambdoidal suture, liquid nitrogen was poured continuously onto the skull for 3 minutes. Thereafter, the brain was dissected onto dry ice to prevent defrosting of the tissue. The excised and frozen brain was stored until further use at −80 °C.

Using a cryostat microtome (Leica Microsystems) set at −21 °C, 12 µm coronal sections (between −0.10 and +0.40 from Bregma) were cut. Consecutive brain sections (3 per brain) were randomly thaw-mounted (in max. 3 seconds) onto indium tin oxide (ITO) coated glass slides (Delta Technologies). In total, 120 sections were analysed with MALDI-MSI. After MALDI-MSI analysis, matrix removal was performed using 70% EtOH where after sections were stained for Nissl substance with Cresyl Violet to enable analysis of the infarct core region.

### SIMS-MSI

All TOF-SIMS analyses were performed using a PHI *nanoTOF* II instrument (Physical Electronics) with a 60 keV Bi_3_^2+^ liquid metal ion gun for MS imaging experiments^37^. The analytical field-of-view (FOV) was in 400 µm × 400 µm with 512 pixels × 512 pixels, resulting in a 0.8 µm pixel size. The sample dose was in all cases at or below the static limit (10^13^ ions cm^−2^). The sample bias was set at 3 kV. The TOF-SIMS (MS^1^) and tandem MS (MS^2^) data were collected simultaneously in positive ion polarity as described in Fisher *et al*.^39^. The tandem MS experiments were performed using 1.5 keV CID with Argon gas estimated to be 1 × 10^−3^ Pa in the collision cell^39^. In the course of each acquisition, mass spectral information at each image pixel was collected in the *m/z* range of 0–3000, and saved into a raw data stream file in both positive and negative polarity.

### MALDI-MSI

Prior to MALDI analysis 2,5-dihydroxybenzoic acid (DHB) and Norharmane matrix were applied to sections at 15 mg/mL and 7 mg/mL in 2:1 chloroform:methanol (v/v), respectively with a TM-sprayer (HTX technologies), using 12 passes at 40 °C, 0.1 mL/min flow rate, 10 psi spray pressure, at 2-mm spray spacing, 1200 mm/min spray velocity, and with a 40-mm sprayer nozzle distance to sample. Consecutive sections were desalted with 50 mM ammonium acetate prior to applying matrix coating. All data was acquired on a Bruker RapifleX MALDI Tissuetyper™ system operating in reflectron mode (Bruker Daltonik GmbH) with a nominal acceleration potential of ±20 kV. MSI data was acquired using a 50 × 50 μm^2^ raster and a 20 × 20 μm^2^ beam scan area for each polarity with 200 laser shots accumulated at each pixel. The average acquisition rate was 23 pixels/second. Data was acquired in the *m/z* range of 400–1000 for positive and 600–1600 for negative ion mode. Mass calibration was performed before each analysis using red phosphorous clusters in positive and negative ion mode. The accurate mass and MS/MS profiles from the tissue sections were acquired on the 9.4 T SolariX FT-ICR (Bruker Daltonik GmbH) and the Synapt G2Si (Waters) in sensitivity mode using 30 eV collision energy.

### Multivariate data analysis

For batch statistical analysis over SIMS-MSI, data was converted into a Matlab matrix by in-house developed software. Once the mean spectrum files were created they were subjected to peak picking using the in-house developed Matlab PEAPI compiled program. The peak list was than integrated over the whole images and Matlab format files were created. The ion images were normalized and reconstructed using HisDistGUI software.

The 2D-datasets were processed using Scils (Bruker Daltonik GmbH) software. The data was TIC-normalised using weak de-noising deterministic installation for both polarities. Automatic peak-picking was performed using the default segmentation pipeline. After acquisition each section was imported into SCilS 2016b. The data was subjected to the segmentation algorithm. Each individual tissue section was annotated and segmented into the three relevant regions of the infarct; core, penumbra and surrounding healthy tissue. The resulting sets of *m/z* values were used to perform pLSA. Ten components were calculated for the negative and seven for the positive ion mode. The components that were representing the matrix distribution were not considered for further evaluation. The generated components gave distinct distributions within the tissue and thereafter each spectrum was presented as a weighted combination of these components. The resulting components and spectra are shown in Fig. 3. Box plots of selected *m/z* values were created in order to see the intensity variations within the selected ROIs of healthy, core and penumbra tissue (n = 3). The mean ratio of signal intensity between ischemic (core, penumbra) and healthy ROIs (n = 3) with the same number of spectra at 4 h, 8 h and 24 h was calculated and expressed as fold increase/decrease with 95% confidence intervals of prominent *m/z* values.

After the components were calculated, the second set of analyses was performed in order to elucidate potential molecular differences between ischemia-relevant areas for wild-type and mutant mice separately. The segmentation algorithm differentiated specific ROIs in each data set. The selected data sets were converted from the Scils (.sl) format into Matlab matrix by an in-house programmed Python script. Once the mean spectrum files were created they were subjected to peak picking with the in-house Matlab PEAPI compiled program. The ROIs were created by the in-house Matlab Trifttricks 1.52 software. On the whole collection of data sets we performed linear discriminant analysis (LDA) displaying different score histograms (Fig. 6) and the corresponding loadings spectra (Supplementary Figs S2 and S3). The histograms are described within the first discriminating function (DF1) revealing the maximum difference within the groups selected. All of the data is described with 80% variance.

## Supplementary information

Supplementary Information

## Data Availability

The datasets generated during and/or analyzed during the current study are available from the corresponding author on reasonable request.

## References

[1] Mozaffarian D (2015). Heart disease and stroke statistics–2015 update: a report from the American Heart Association. Circulation..

[2] Hossmann KA (1994). Viability thresholds and the penumbra of focal ischemia. Ann Neurol..

[3] Hankin JA (2011). MALDI mass spectrometric imaging of lipids in rat brain injury models. J Am Soc Mass Spectrom..

[4] Janfelt C (2012). Visualization by mass spectrometry of 2-dimensional changes in rat brain lipids, including N-acylphosphatidylethanolamines, during neonatal brain ischemia. FASEB J..

[5] Koizumi S (2010). Imaging mass spectrometry revealed the production of lyso-phosphatidylcholine in the injured ischemic rat brain. Neuroscience..

[6] Shanta SR (2012). Global changes in phospholipids identified by MALDI MS in rats with focal cerebral ischemia. J Lipid Res..

[7] Whitehead SN (2011). Imaging mass spectrometry detection of gangliosides species in the mouse brain following transient focal cerebral ischemia and long-term recovery. PLoS One..

[8] Goodwin RJ, Iverson SL, Andren PE (2012). The significance of ambient-temperature on pharmaceutical and endogenous compound abundance and distribution in tissues sections when analyzed by matrix-assisted laser desorption/ionization mass spectrometry imaging. Rapid Commun Mass Spectrom..

[9] Sugiura Y, Honda K, Kajimura M, Suematsu M (2014). Visualization and quantification of cerebral metabolic fluxes of glucose in awake mice. Proteomics..

[10] Braidy N (2014). Metal and complementary molecular bioimaging in Alzheimer’s disease. Front Aging Neurosci.

[11] Carlred L (2016). Probing amyloid-beta pathology in transgenic Alzheimer’s disease (tgArcSwe) mice using MALDI imaging mass spectrometry. J Neurochem..

[12] Hanrieder, J., Ljungdahl, A. & Andersson, M. MALDI imaging mass spectrometry of neuropeptides in Parkinson’s disease. *J Vis Exp*. **60** (2012). 10.3791/3445 PMC3529518 22370902

[13] Hong JH (2016). Global changes of phospholipids identified by MALDI imaging mass spectrometry in a mouse model of Alzheimer’s disease. J Lipid Res..

[14] Wang HY, Wu HW, Tsai PJ, Liu CB (2012). MALDI-mass spectrometry imaging of desalted rat brain sections reveals ischemia-mediated changes of lipids. Anal Bioanal Chem..

[15] Lanekoff I, Stevens SL, Stenzel-Poore MP, Laskin J (2014). Matrix effects in biological mass spectrometry imaging: identification and compensation. Analyst..

[16] van den Maagdenberg AM (2004). A Cacna1a knockin migraine mouse model with increased susceptibility to cortical spreading depression. Neuron..

[17] Milne SB, Ivanova PT, DeCamp D, Hsueh RC, Brown HA (2005). A targeted mass spectrometric analysis of phosphatidylinositol phosphate species. J Lipid Res..

[18] Hsu FF, Turka J (2000). Characterization of phosphatidylinositol, phosphatidylinositol-4-phosphate, and phosphatidylinositol-4,5-bisphosphate by electrospray ionization tandem mass spectrometry: a mechanistic study. J Am Soc Mass Spectrom..

[19] Paradies G, Petrosillo G, Paradies V, Ruggiero FM (2009). Role of cardiolipin peroxidation and Ca2+ in mitochondrial dysfunction and disease. Cell Calcium..

[20] Sugiura Y, Shimma S, Konishi Y, Yamada MK, Setou M (2008). Imaging mass spectrometry technology and application on ganglioside study; visualization of age-dependent accumulation of C20-ganglioside molecular species in the mouse hippocampus. PLoS One..

[21] Eikermann-Haerter K (2012). Migraine mutations increase stroke vulnerability by facilitating ischemic depolarizations. Circulation..

[22] Dirnagl U, Iadecola C, Moskowitz MA (1999). Pathobiology of ischaemic stroke: an integrated view. Trends Neurosci..

[23] Abe K, Kogure K, Yamamoto H, Imazawa M, Miyamoto K (1987). Mechanism of arachidonic acid liberation during ischemia in gerbil cerebral cortex. J Neurochem..

[24] Huang SF, Sun GY (1986). Cerebral ischemia induced quantitative changes in rat brain membrane lipids involved in phosphoinositide metabolism. Neurochem Int..

[25] Ikeda M, Yoshida S, Busto R, Santiso M, Ginsberg MD (1986). Polyphosphoinositides as a probable source of brain free fatty acids accumulated at the onset of ischemia. J Neurochem..

[26] Lin TN, Liu TH, Xu J, Hsu CY, Sun GY (1991). Brain polyphosphoinositide metabolism during focal ischemia in rat cortex. Stroke..

[27] Sun GY, Huang HM, Chandrasekhar R (1988). Turnover of inositol phosphates in brain during ischemia-induced breakdown of polyphosphoinositides. Neurochem Int..

[28] Sun GY (1995). Inositol trisphosphate, polyphosphoinositide turnover, and high-energy metabolites in focal cerebral ischemia and reperfusion. Stroke..

[29] Amoscato AA (2014). Imaging mass spectrometry of diversified cardiolipin molecular species in the brain. Anal Chem..

[30] Roux A (2016). Mass spectrometry imaging of rat brain lipid profile changes over time following traumatic brain injury. J Neurosci Methods..

[31] Sparvero LJ (2016). Imaging mass spectrometry reveals loss of polyunsaturated cardiolipins in the cortical contusion, hippocampus, and thalamus after traumatic brain injury. J Neurochem..

[32] Yoon JS (2018). Spatiotemporal protein atlas of cell death-related molecules in the rat MCAO stroke model. Exp Neurobiol..

[33] Adibhatla RM, Hatcher JF, Dempsey RJ (2006). Lipids and lipidomics in brain injury and diseases. AAPS J..

[34] Yu RK, Tsai YT, Ariga T, Yanagisawa M (2011). Structures, biosynthesis, and functions of gangliosides - an overview. J Oleo Sci..

[35] Karpiak SE, Li YS, Mahadik SP (1987). Gangliosides (GM1 and AGF2) reduce mortality due to ischemia: protection of membrane function. Stroke..

[36] Tanaka K (1986). Effect of the ganglioside GM1, on cerebral metabolism, microcirculation, recovery kinetics of ECoG and histology, during the recovery period following focal ischemia in cats. Stroke..

[37] Longa EZ, Weinstein PR, Carlson S, Cummins R (1989). Reversible middle cerebral artery occlusion without craniectomy in rats. Stroke..

[38] Mulder IA (2016). Funnel-freezing versus heat-stabilization for the visualization of metabolites by mass spectrometry imaging in a mouse stroke model. Proteomics..

[39] Fisher GL, Hammond JS, Bryan SR, Larson PE, Heeren RMA (2017). The Composition of Poly(Ethylene Terephthalate) (PET) Surface Precipitates Determined at High Resolving Power by Tandem Mass Spectrometry Imaging. Microsc Microanal..

