# Supplementary material for: Distinguishing core from penumbra by lipid profiles using Mass Spectrometry Imaging in a transgenic mouse model of ischemic stroke
Source: Sci Rep. 2019 Jan 31;9:1090. doi: 10.1038/s41598-018-37612-5 (PMC6355923; doi:10.1038/s41598-018-37612-5)
Supplement: Supplementary file 1 — Supplementary Information [file 41598_2018_37612_MOESM1_ESM.pdf]

Supplementary Information for

Distinguishing core from penumbra by lipid profiles using Mass Spectrometry Imaging in a transgenic mouse model of ischemic stroke

I.A. Mulder, N. Ogrinc Potočnik, L.A.M. Broos, A. Prop, M.J.H. Wermer, R.M.A. Heeren and A.M.J.M. van den Maagdenberg

Arn M.J.M. van den Maagdenberg

E-mail: A.M.J.M.van_den_Maagdenberg@lumc.nl

This PDF file includes:

Figs. S1 to S8

Tables S1 and S2

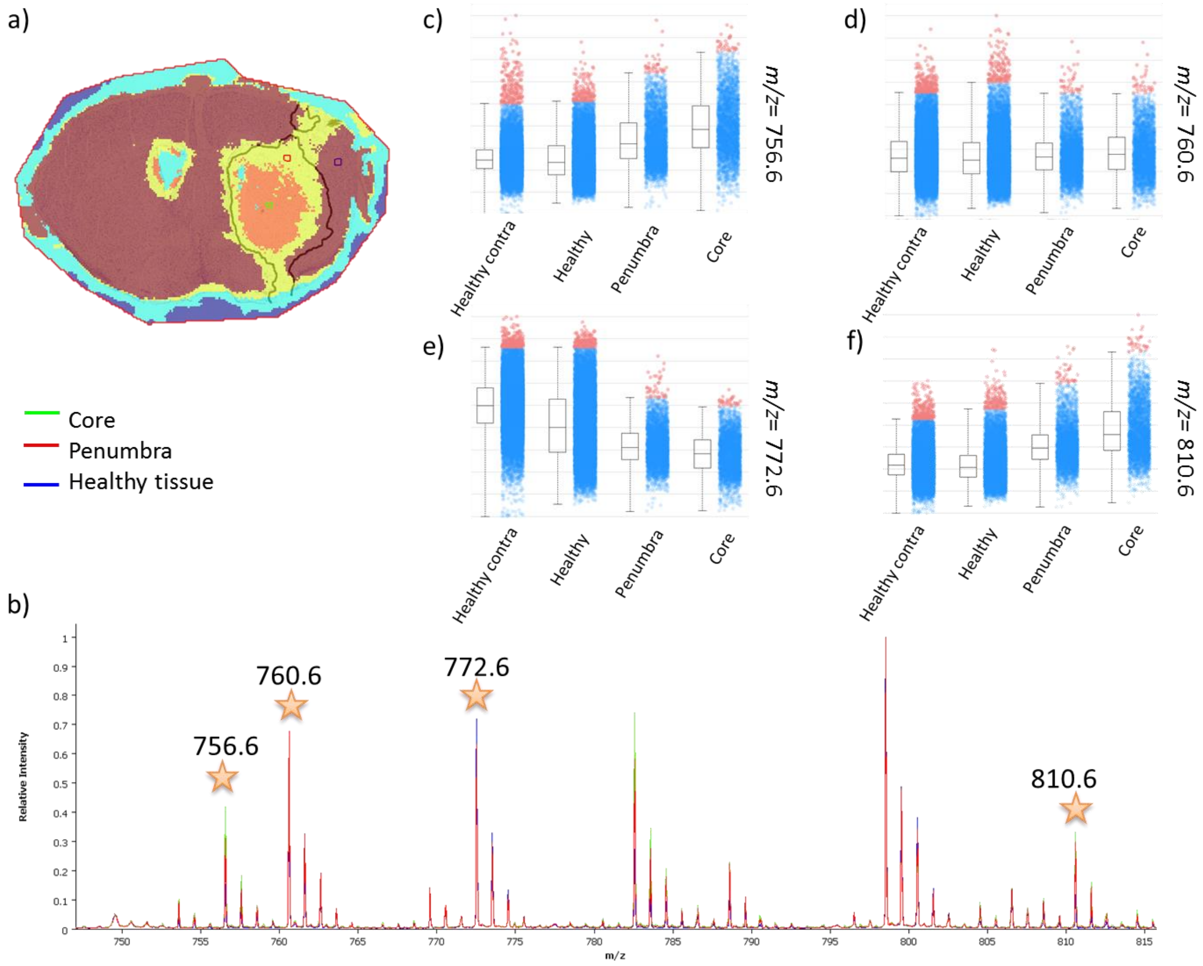

Fig. S1. Positive ion mode mean spectra of ROI regions selected from the segmented core (green), penumbra (red), and healthy tissue (blue) with the same average number of spectra. The starred *m/z*=756.6 [PC(32:0)+Na]$^+$, 760.6 [PC(32:0)+H]$^+$, 772.6 [PC(36:0)+K]$^+$ and 810.6 [PC(36:1)+Na]$^+$ show an increase/decrease or no changes in relative intensities in the core region. Their box intensity plots (n=3) c) *m/z*=756.6 [PC(32:0)+Na]$^+$, d) 760.6 [PC(32:0)+H]$^+$ e) 772.6 [PC(36:0)+K]$^+$ and f) 810.6 [PC(36:1)+Na]$^+$ show the intensity variations through the selected tissue areas: healthy contra, healthy, penumbra and core at 4 hours post MCAO.

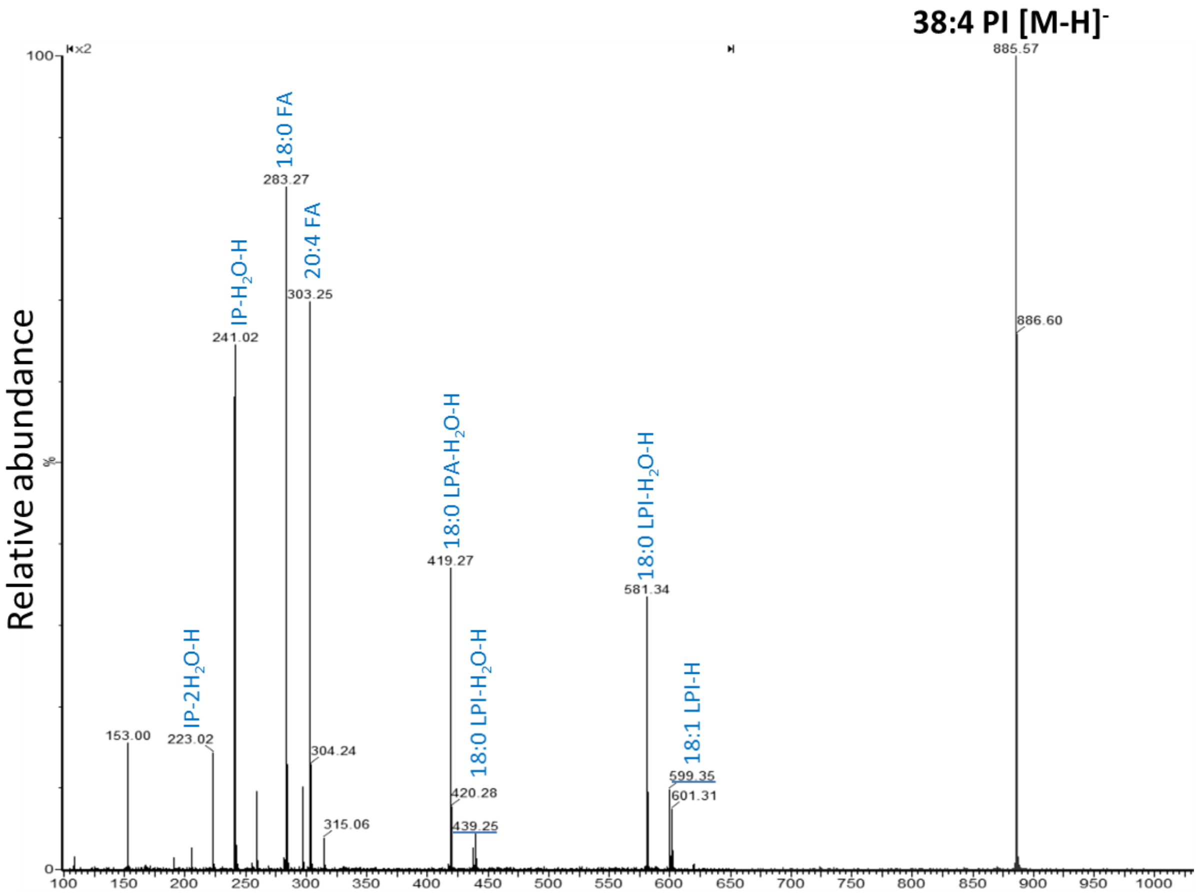

Fig. S2. MS/MS negative ion mode spectrum of phosphatidylinositol 4-phosphate PI(38:4) [M-H]$^-$ with precursor mass *m/z*=885.5 and associated annotated fragments.

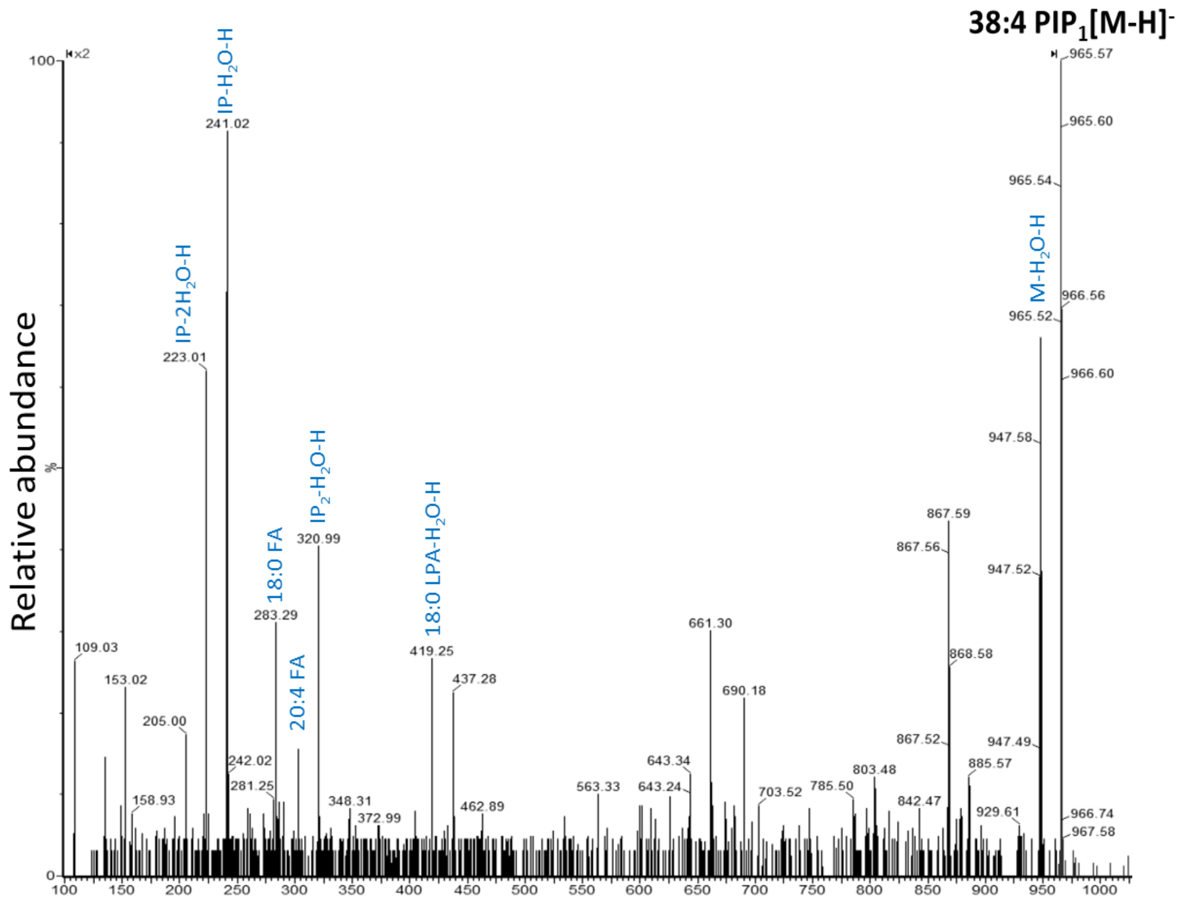

Fig. S3. MS/MS negative ion mode spectrum spectrum of phosphatidylinositol 4-bisphosphate (PIP_1) (38:4) $[M-H]^-$ with precursor mass *m/z*=965.5 and associated fragments.

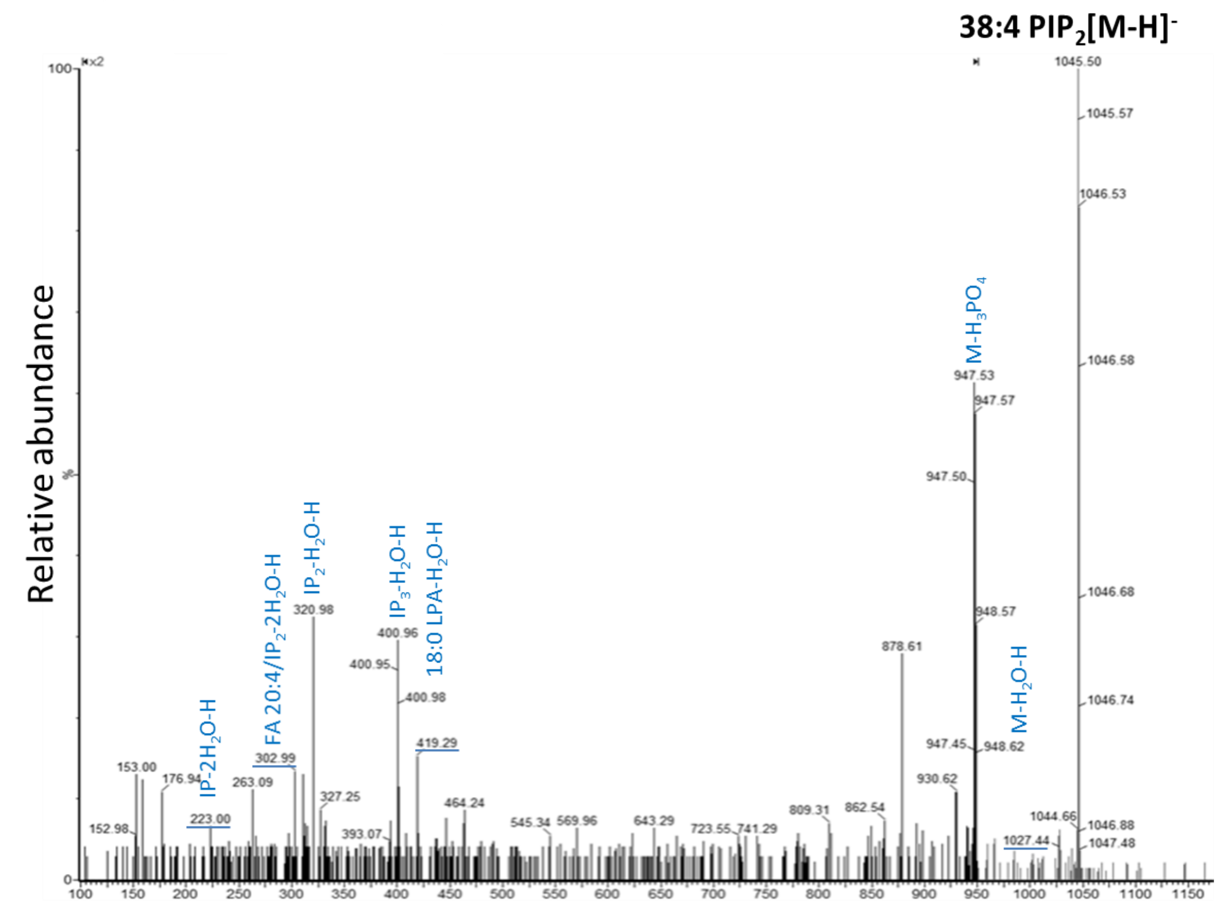

Fig. S4. MS/MS negative ion mode spectrum of phosphatidylinositol 4-5 -bisphosphate (PIP_2) (38:4) $[M-H]^-$ with precursor mass *m/z*=1045.5 and associated fragments.

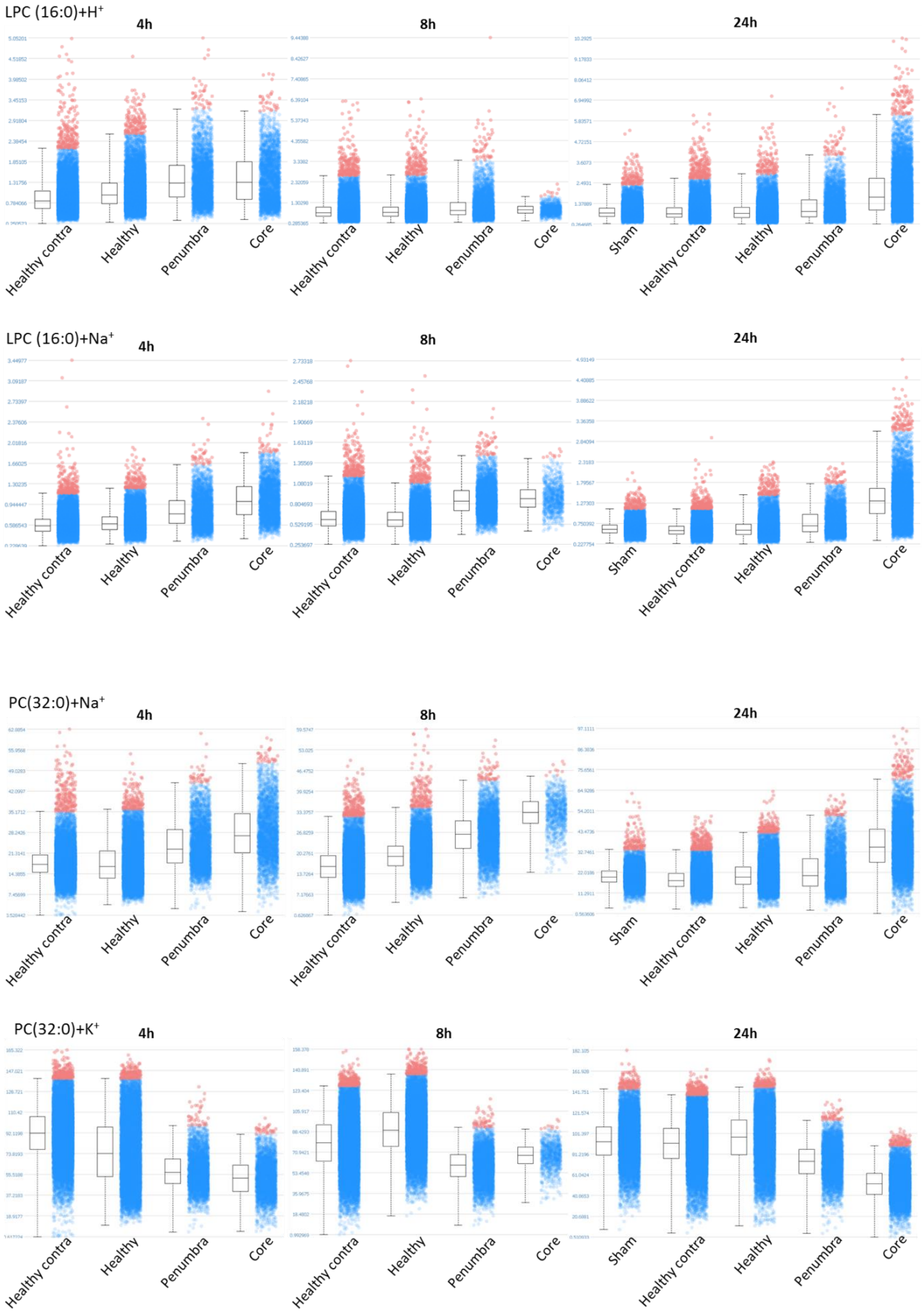
LPC (16:0)+H+
4h
8h
24h
Healthy contra
Healthy
Penumbra
Core
Sham
LPC (16:0)+Na+
PC(32:0)+Na+
PC(32:0)+K+

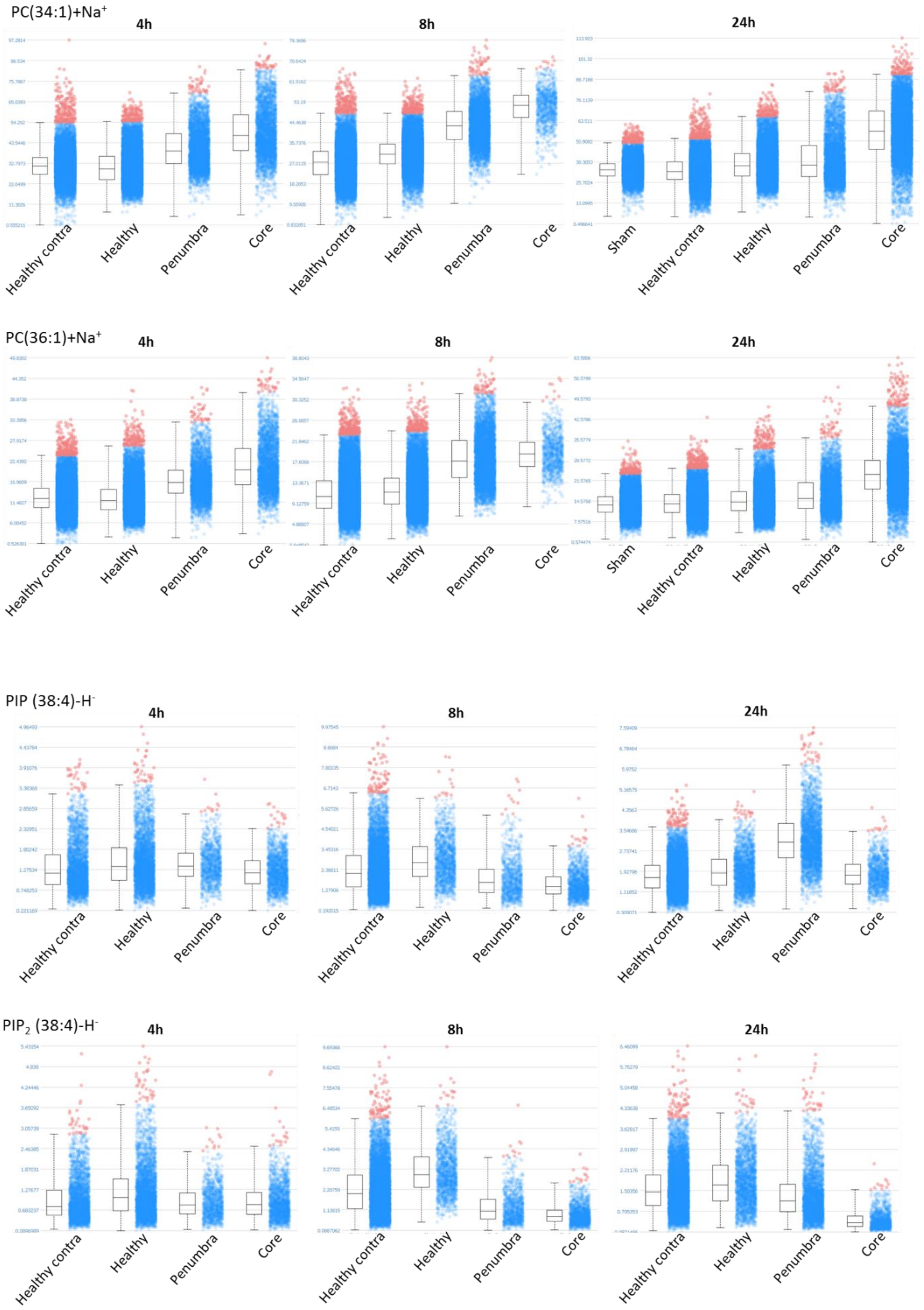
PC(34:1)+Na+
4h
8h
24h
Healthy contra
Healthy
Penumbra
Core
Sham
PC(36:1)+Na+
4h
8h
24h
PIP (38:4)-H-
4h
8h
24h
PIP2 (38:4)-H-
4h
8h
24h

Fig. S5. Box plots of selected ions (LPC (16:0)+H^+, LPC (16:0)+ Na^+, PC(32:0)+Na^+, PC(32:0)+K^+ PC(34:1)+Na^+ PC(36:1)+Na^+) of their intensity variations within the selected ROIs of healthy contra, healthy, core and penumbra tissue at 4 and 8 hours, and including sham at 24 hours in positive ion mode (n=3). Box plots of selected ions (PIP (38:4)-H^- and PIP2 (38:4) -H^-) of their intensity variations within the selected ROIs of healthy contra, healthy, core and penumbra tissue at 4, 8h, 24 hours in negative ion mode (n=2).

Table S1. Washed and unwashed MALDI images in positive ion mode at 4, 8 and 24 hours in wild-type tissue after transient middle cerebral artery occlusion.

m/z value	Compound	SHAM	4 hours	8 hours	24 hours
Nissl stain		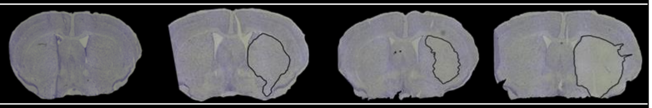			
756.6	[PC(32:0)+Na]$^+$	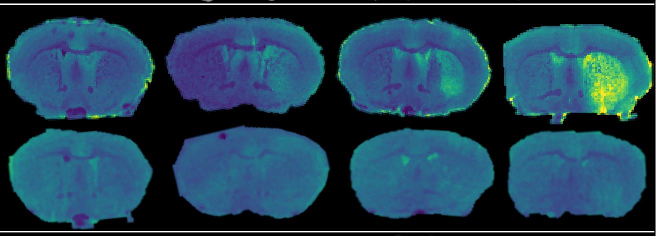			
760.6	[PC(34:1)+H]$^+$	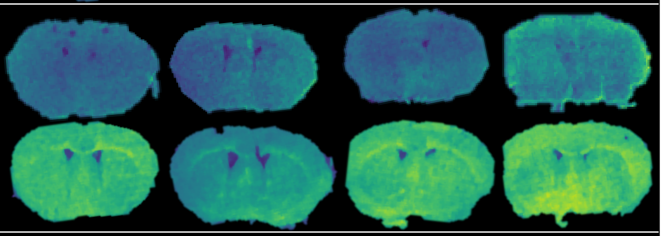			
769.6	[SM(18:0)+K]$^+$	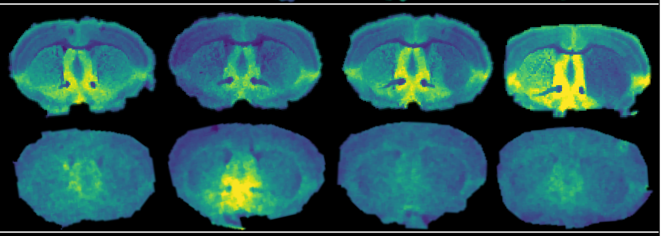			
772.6	[PC(32:0)+K]$^+$	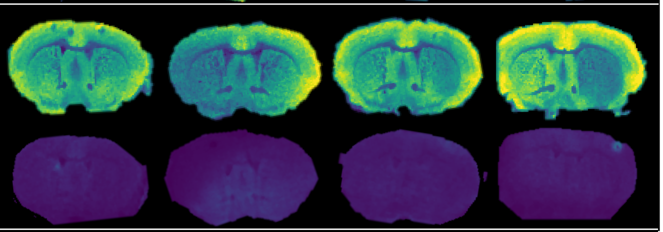			
782.6	[PC(34:1)+Na]+	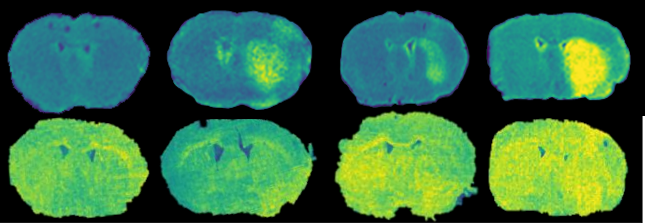			
788.6	[PC(36:1)+H]$^+$	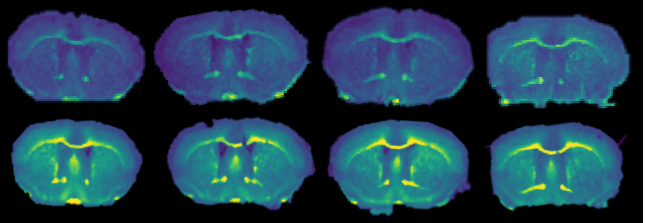			
798.6	[PC(34:1)+K]$^+$	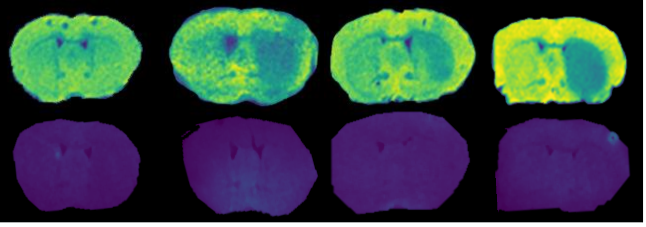			

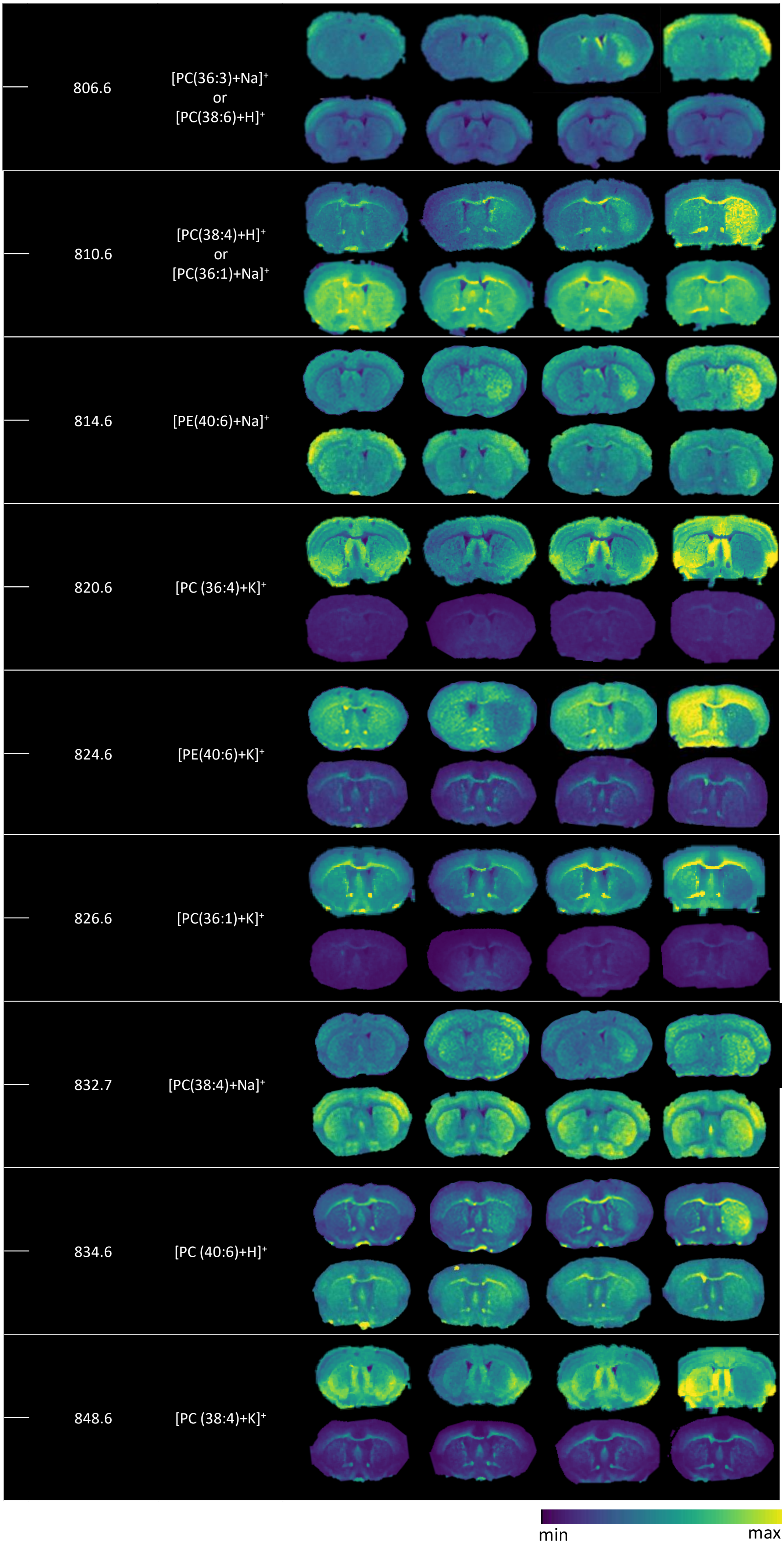
806.6
[PC(36:3)+Na]+ or [PC(38:6)+H]+
810.6
[PC(38:4)+H]+ or [PC(36:1)+Na]+
814.6
[PE(40:6)+Na]+
820.6
[PC (36:4)+K]+
824.6
[PE(40:6)+K]+
826.6
[PC(36:1)+K]+
832.7
[PC(38:4)+Na]+
834.6
[PC (40:6)+H]+
848.6
[PC (38:4)+K]+
min
max

Selected MALDI *m/z* images acquired at 50-µm spatial resolution showing the distribution of selected lipid phosphatidylcholine (PC) species of washed (top row) and un-washed (bottom row) wild-type (WT) tissue sections at 4, 8 and 24 hours after transient middle cerebral artery occlusion in positive ion mode.

Table S2. Distribution of cardiolipin and ganglioside species in wild-type tissue sections.

m/z value	Compound	4 hours	8 hours	24 hours
		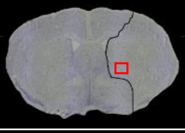	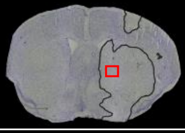	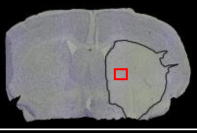
		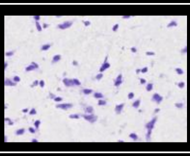	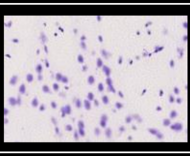	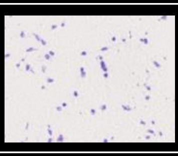
1452.3	CL(72:6) $[M-H]^-$	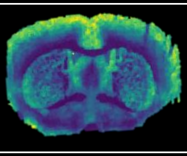	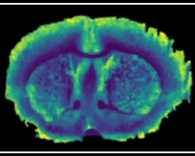	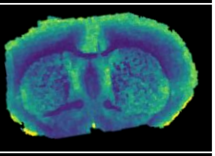
1476.3	CL(74:8) $[M-H]^-$	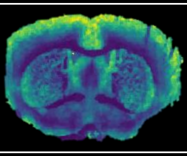	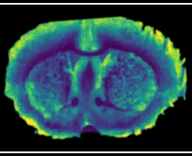	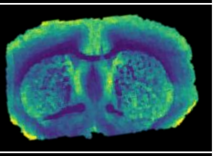
1497.3	CL(76:11) $[M-H]^-$	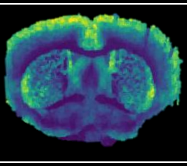	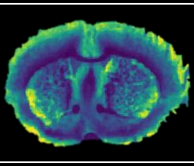	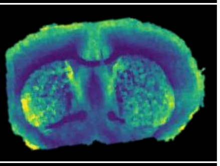
1544.6	GM1(18:0) $[M-H]^-$	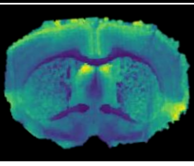	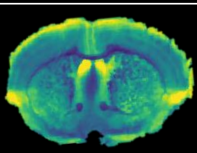	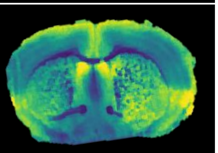

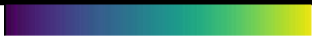

Selected MALDI *m/z* images acquired at 50-µm spatial resolution showing the distribution of cardiolipin (CL) and ganglioside (GM) $[M-H]^-$species in wild-type tissue at 4, 8 and 24 hours after transient middle cerebral artery occlusion in negative ion mode.

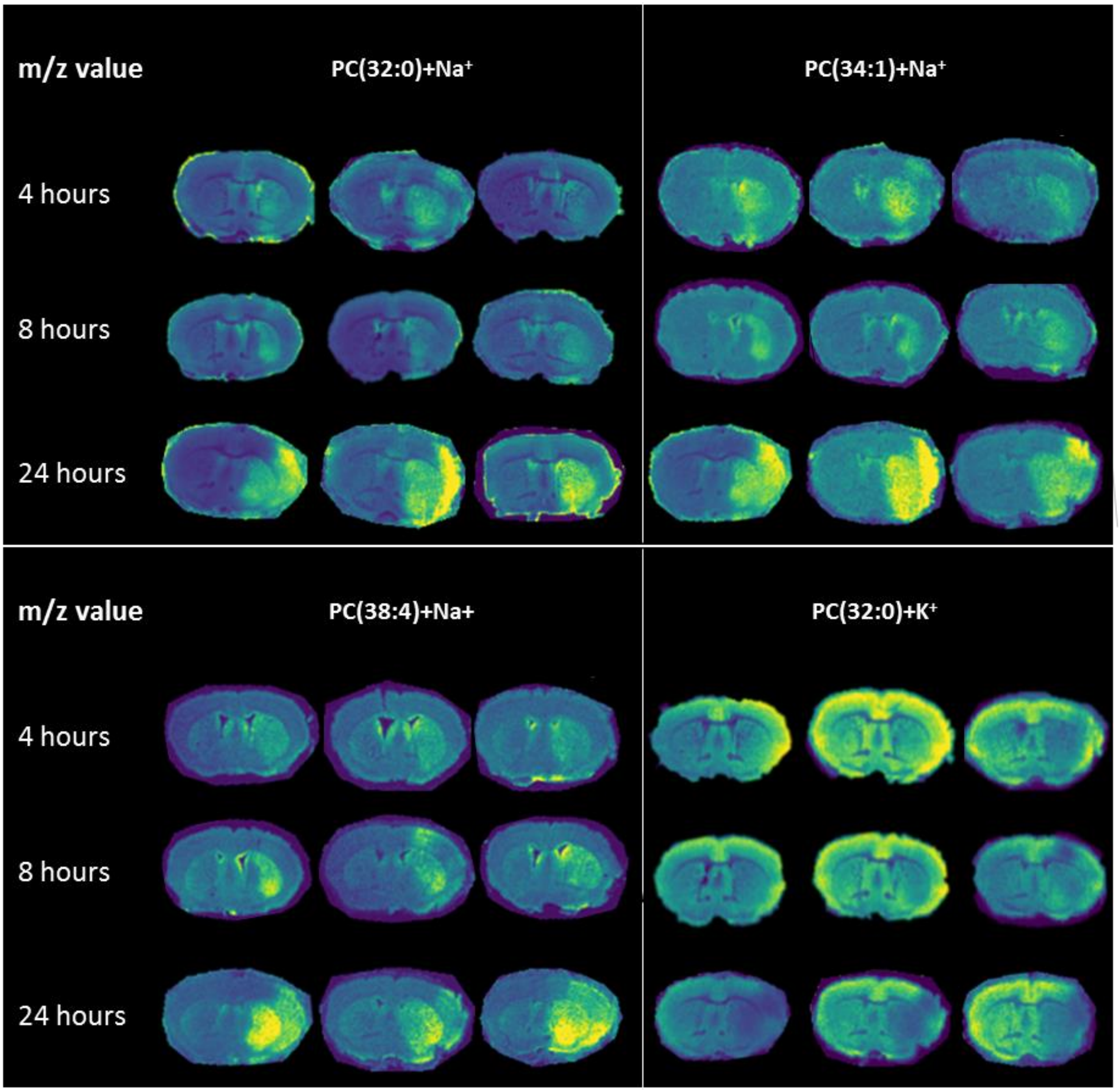

Fig. S6. Examples of biological replicas (n=3) of PC(32:0)+Na^+, PC(34:1)+$Na^{+,}$ PC(38:0)+Na^+, PC(32:0)+K^+ at different timepoints (4, 8 and 24 hours) post MCAO.

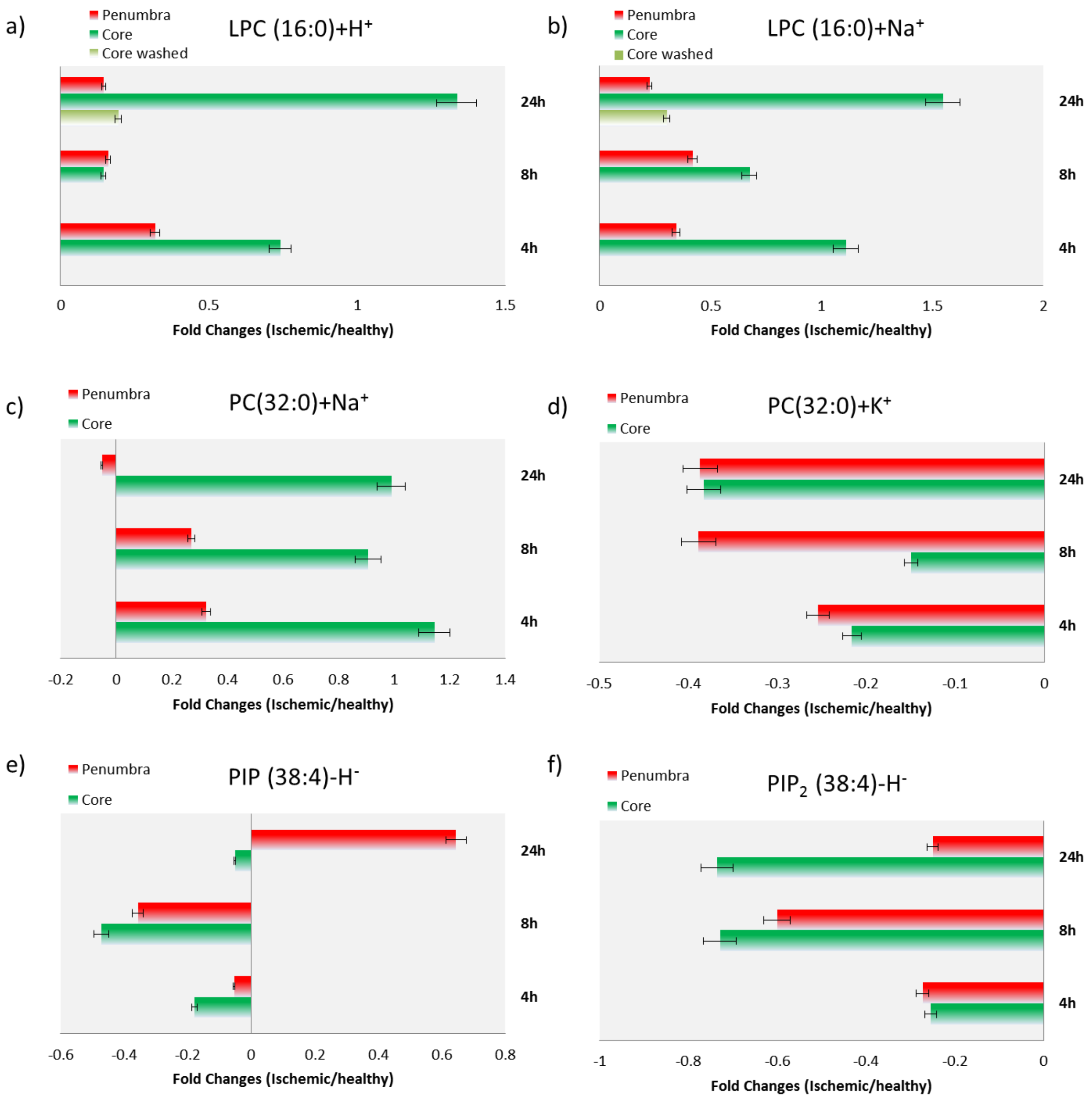

Fig. S7. Data represents the fold changes expressed as the mean ratio of signal intensity between ischemic (core, penumbra) and healthy ROIs (n=3) at 4, 8 and 24 hours after induced MCAO. Fold changes with 5% error were expressed for the following ions a) LPC (16:0)+H+ m/z=496.4, b) LPC (16:0)+ Na+ m/z= 518.5, c) [PC(32:0)+Na]+ m/z= 756.6, [PC(32:0)+K]+ in positive ion mode and e) PIP (38:4)-H- and PIP2 (38:4)-H- in negative ion mode. All species showed statistical significants p<0.05.

a)

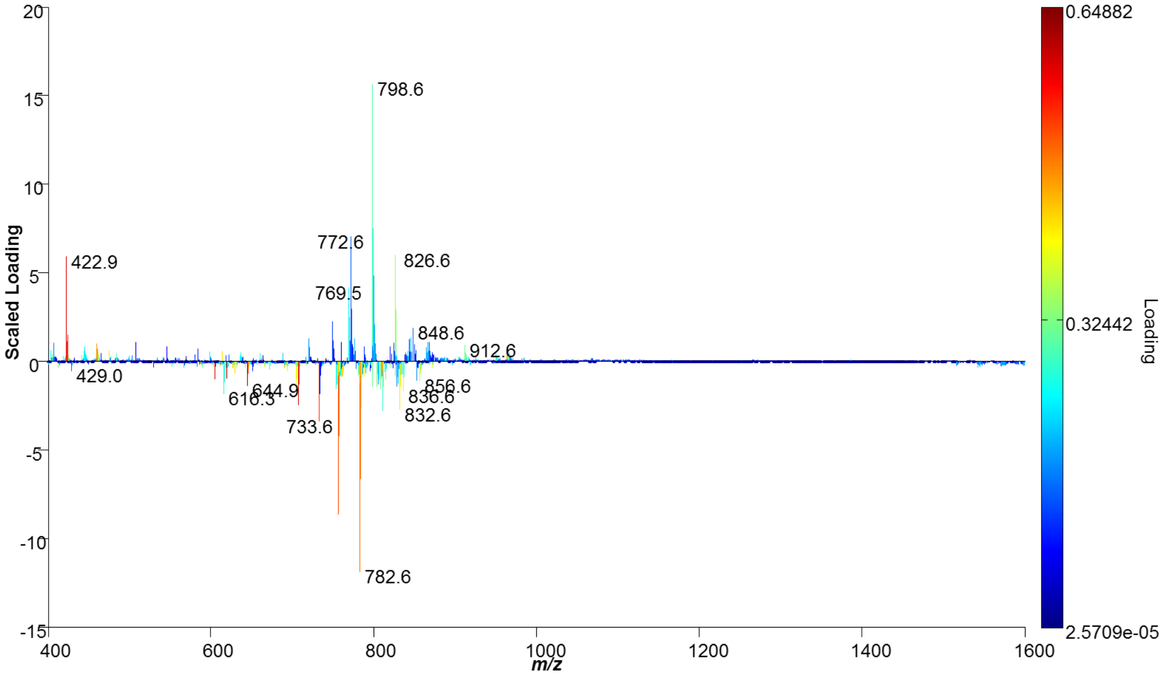

b)

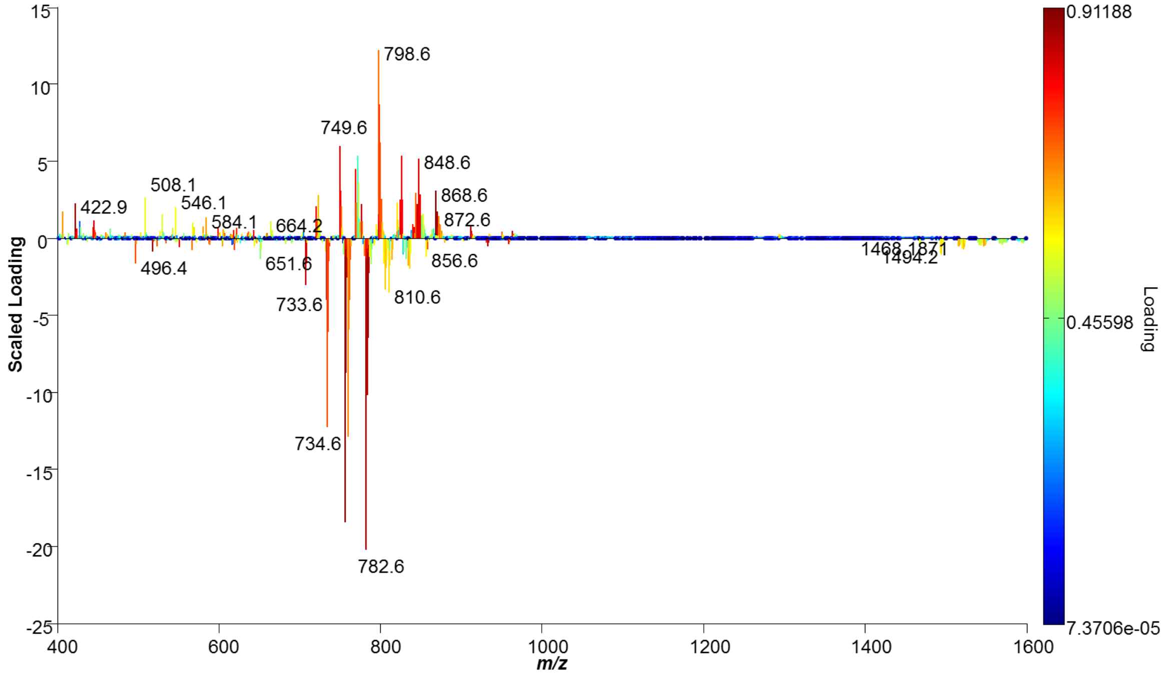

Fig. S8. Loadings spectra of the histograms at 24 hours of a) wild-type and b) FHM1 mutant tissue. The colour bars indicate the contribution of individual masses to the positive or negative loadings. High contribution in red and low contribution in dark blue.
